# Prevalence and Distribution of Potentially Human Pathogenic *Vibrio* spp. on German North and Baltic Sea Coasts

**DOI:** 10.3389/fcimb.2022.846819

**Published:** 2022-07-22

**Authors:** Susanne Fleischmann, Ilona Herrig, Jessica Wesp, Joscha Stiedl, Georg Reifferscheid, Eckhard Strauch, Thomas Alter, Nicole Brennholt

**Affiliations:** ^1^ Department G3-Bio-Chemistry, Ecotoxicology, Federal Institute of Hydrology, Koblenz, Germany; ^2^ School of Veterinary Medicine, Institute of Food Safety and Food Hygiene, Freie Universität Berlin, Berlin, Germany; ^3^ Department of Biological Safety, German Federal Institute for Risk Assessment, Berlin, Germany

**Keywords:** prevalence, human pathogen, climate-related infectious diseases, *Vibrio* spp., German coastal waters

## Abstract

Global ocean warming results in an increase of infectious diseases including an elevated emergence of *Vibrio* spp. in Northern Europe. The European Centre for Disease Prevention and Control reported annual periods of high to very high risks of infection with *Vibrio* spp. during summer months along the North Sea and Baltic Sea coasts. Based on those facts, the risk of *Vibrio* infections associated with recreational bathing in European coastal waters increases. To obtain an overview of the seasonal and spatial distribution of potentially human pathogenic *Vibrio* spp. at German coasts, this study monitored *V. cholerae*, *V. parahaemolyticus*, and *V. vulnificus* at seven recreational bathing areas from 2017 to 2018, including the heat wave event in summer 2018. The study shows that all three *Vibrio* species occurred in water and sediment samples at all sampling sites. Temperature was shown to be the main driving factor of *Vibrio* abundance, whereas *Vibrio* community composition was mainly modulated by salinity. A species-specific rapid increase was observed at water temperatures above 10°C, reaching the highest detection numbers during the heat wave event with abundances of 4.5 log10 CFU+1/100 ml of seawater and 6.5 log10 CFU+1/100 g of sediment. Due to salinity, the dominant *Vibrio* species found in North Sea samples was *V. parahaemolyticus*, whereas *V. vulnificus* was predominantly detected in Baltic Sea samples. Most detections of *V. cholerae* were associated with estuarine samples from both seas. *Vibrio* spp. concentrations in sediments were up to three log higher compared to water samples, indicating that sediments are an important habitat for *Vibrio* spp. to persist in the environment. Antibiotic resistances were found against beta-lactam antibiotics (ampicillin 31%, cefazolin 36%, and oxacillin and penicillin 100%) and trimethoprim-sulfamethoxazole (45%). Moreover, isolates harboring pathogenicity-associated genes such as *trh* for *V. parahaemolyticus* as well as *vcg*, *cap*/*wcv*, and the 16S rRNA-type B variant for *V. vulnificus* were detected. All sampled *V. cholerae* isolates were identified as non-toxigenic non-O1/non-O139 serotypes. To sum up, increasing water temperatures at German North Sea and Baltic Sea coasts provoke elevated *Vibrio* numbers and encourage human recreational water activities, resulting in increased exposure rates. Owing to a moderate Baltic Sea salinity, the risk of *V. vulnificus* infections is of particular concern.

## Introduction

*Vibrio* (*V*.) species occur naturally in marine and estuarine aquatic ecosystems and are ubiquitously distributed worldwide. Next to over 100 identified *Vibrio* species, three *Vibrio* species are strongly associated with human infections (Baker-Austin et al., 2018): (1) *V. cholerae* serogroups O1 or O139 are an important cause of diarrheal disease in many countries worldwide. The pathogenicity of these pandemic serogroups is mainly attributed to the cholera toxin, a potent enterotoxin, and the toxin-coregulated pilus, essential for intestinal colonization. However, recent studies indicate that non-O1 or non-O139 *V. cholerae* can also cause cholera-like diarrheal diseases, even though they do not encode the main cholera virulence factors. In addition, a direct ear, eye, and wound contact can lead to extraintestinal infections including septicemia (Shin et al., 2011; Deshayes et al., 2015; Chowdhury et al., 2016; Baker-Austin et al., 2018). (2) *V. parahaemolyticus* is mainly associated with gastrointestinal infections through the consumption of raw or undercooked seafood. Nevertheless, similar to *V. cholerae*, *V. parahaemolyticus* can cause extraintestinal infections through direct contact with open wounds leading to severe infections (Baker-Austin et al., 2018; Yang et al., 2019; Guillod et al., 2019). (3) Finally, *V. vulnificus* can also cause a seafood-borne gastroenteritis but is mainly associated with wound infections by direct wound or skin lesion contact. Severe infections can lead to amputations of the affected limbs, and septicemia often results in fatal multiple organ failure. With respect to 459 infections with *V. vulnificus* reported by the Food and Drug Administration (FDA) in the US between 1992 and 2007, 51.6% of the infected patients died (Jones and Oliver, 2009; Baker-Austin and Oliver, 2018; Bhat et al., 2019; Guillod et al., 2019; Leng et al., 2019). With the exception of pandemic O1 and O139 *V. cholerae*, the three mentioned potentially human pathogenic *Vibrio* species have been isolated from seafood and coastal waters around the world (Pruzzo et al., 2005).

The Intergovernmental Panel on Climate Change (IPCC) has documented ocean warming and an increase in sea levels because of climate change worldwide. Changes in geographic and seasonal weather patterns resulted in extreme weather and climate events such as heatwaves, violent storms, and heavy rainfall leading to flooding events. The result is an intensification of climate-sensitive infectious diseases with increasing morbidity and mortality including an elevated risk of infection with *Vibrio* spp. in northern Europe (IPCC, 2019). Due to their ability to replicate in 20 min or less in optimal conditions, extreme weather conditions can lead to so-called *Vibrio*-blooms. Since 1970 (Kaneko and Colwell, 1973), numerous studies have shown that temperature, besides nutrient and salt content, is the primary factor to influence the *Vibrio* spp. occurrence in temperate waters (Baker-Austin et al., 2013; Vezzulli et al., 2013; Baker-Austin et al., 2018; Froelich and Daines, 2020).

From January 2010 to September 2020, a warming trend of the average land surface temperature of 1.2°C has been observed in Europe. From a global perspective, the average decadal land surface temperature has risen by 0.57°C, while the ocean surface temperature has increased by 0.33°C in the last 10 years. During this time, the longest sustained heat wave in Europe started in April 2018 at 3.0°C and ended in August 2018 1.9°C above the average temperature of the 20th century. In that year, the annual average land surface temperature was 1.9°C above the average temperature of the 20th century (NOAA, 2010-2020). The surface water temperatures of the North Sea and the Baltic Sea in July 2018 were 2.0°C and 2.8°C above the average temperature for July in the 20th century, respectively (BSH, 2020). In the last decade, parallel to the warming trend of the North Sea and Baltic Sea, an increase in *Vibrio* infection cases has been observed (Baker-Austin et al., 2010; Baker-Austin et al., 2013; Vezzulli et al., 2016; Brehm et al., 2021). Simultaneously since 2013, the European Centre for Disease Prevention and Control annually reported a high to very high infection risk along the European coast during the summer period (ECDC 2013–2020). Heat waves resulted in increased infection numbers in Sweden and Finland in summer 2014. A total of 89 infection cases with *Vibrio* spp. were reported (Baker-Austin et al., 2016; Baker-Austin et al., 2017). During the heat wave in 2018, three deaths associated with pre-existing chronic diseases and 17 *Vibrio* infections were registered by the competent health authorities in Germany. Additional investigations by Brehm et al. (2021) resulted in a total number of 47 cases attributed to *Vibrio* infections. All infection cases were related to direct water contact through bathing in the German North Sea and Baltic Sea. In the previous years, less infections were reported, only one infection in 2017, three infections in 2016, and eight infections in 2015. Since 2014, the State Agency for Health and Social Affairs Mecklenburg-Western Pomerania (LAGuS) has released annual reports about an increased infection risk due to high water temperatures during the bathing season in comparison to the evidence of *Vibrio* spp. at selected bathing areas on the Baltic coast in Mecklenburg-Western Pomerania. Ongoing warm water temperatures in 2019 also caused 34 *Vibrio* infections and two deaths on the German coasts (LAGuS, 2014-2020; Brehm et al., 2021).

Besides these facts, increasing water temperatures provoke an increase in human activity in bathing waters as well as the abundance of waterborne bacteria like *Vibrio* species, resulting in an elevated infection potential (Froelich and Daines, 2020; Vezzulli et al., 2020). In particular, children, pregnant women, older adults, and immunocompromised people with chronic diseases or people who take certain medications belong to the population with the highest risk of infections (IPCC, 2019). A case report of a 31-year-old freshly tattooed man who died from a *V. vulnificus* infection after bathing in the Gulf of Mexico in 2017 demonstrated that even tiny wounds are sufficient to facilitate the entrance for *Vibrio* spp. causing severe wound infections (Hendren et al., 2017). Infections with *Vibrio* spp. show a peak between May and October (CDC, 2017). However, the period of a higher infection risk expands at the German coasts because of warmer winter periods and ongoing warm temperatures. Therefore, the temperature-dependent increase in *Vibrio* spp. begins early in spring, reaches the highest concentrations in summer, and does not end until late autumn. Indeed, human pathogenic *V. cholerae*, *V. parahaemolyticus*, and *V. vulnificus* have an optimal growth temperature between 30 and 40°C, but the risk of infections starts with a bacterial increase at temperatures from 13 to 18°C (Martinez-Urtaza et al., 2010; Oliver et al., 2013; Baker-Austin and Oliver, 2018).

Next to rising water temperatures, salinity is another important parameter with a significant impact on the composition of the *Vibrio* spp. community. *V. cholerae*, *V. parahaemolyticus*, and *V. vulnificus* especially differ in their salinity requirements and tolerances. *V. cholerae* is often associated with contaminated (drinking) water and can persist in freshwater, whereas *V. parahaemolyticus* and *V. vulnificus* are adapted to salt contents present in coastal waters. The salt concentration for an optimal growth of *V. parahaemolyticus* is between 10‰ and 34‰, and its salt tolerance is higher than that of *V. vulnificus*, which is limited to approximately 25‰ (Randa et al., 2004; Froelich et al., 2015; Baker-Austin et al., 2018). However, temperature and salinity are responsible for influencing more than 50% of the *Vibrio* spp. composition (Wetz et al., 2008; Nigro et al., 2011; Froelich et al., 2013; Froelich et al., 2019; Froelich and Daines, 2020). Therefore, infections with *Vibrio* spp. are linked to warmer water temperatures above approximately 15°C and a moderate salinity between 2‰ and 25‰. Estuaries and coastal floodwaters have such brackish water conditions (Vezzulli et al., 2013; Froelich et al., 2015; Baker-Austin et al., 2018; Froelich and Daines, 2020).

In response to all these facts and in order to estimate the situation on the German coast, especially during hot-summer periods, the present study monitored the seasonal and spatial distribution of potentially human pathogenic *Vibrio* spp. from seven recreational bathing areas of the North Sea and the Baltic Sea. Therefore, samples were taken over a period of 14 to 16 months in relation to environmental conditions. *V. cholerae*, *V. parahaemolyticus*, and *V. vulnificus* were quantified monthly from sediment and water samples, using culture-based and quantitative real-time PCR assays. Species-specific gene targets as well as mass spectrometry were used for species identification. The pathogenic potential of all collected isolates was also studied *via* PCR using specific gene targets for selected pathogenicity-associated genes. Furthermore, antibiotic resistance for selected antibiotics was tested using the disk diffusion method. Based on the findings of these investigations, the following research questions were addressed: (i) How do environmental conditions such as water temperature and salinity influence the distribution patterns of potential human pathogenic *Vibrio* spp. of recreational bathing areas at the German North Sea and Baltic Sea? (ii) Are virulent and antibiotic-resistant strains present in the *Vibrio* community?

## Materials and Methods

### Study Area

In total, seven coastal recreational bathing areas in Germany were monitored for the occurrence of potentially pathogenic *Vibrio* species. Three sampling sites were located at the North Sea in Lower Saxony and four at the Baltic Sea in Mecklenburg-Western Pomerania. Sites were selected to comprise different salinity ranges according to Annex II of the European Water Framework Directive for classification of coastal waters (**Table 1**). The coastal water typologies at the sampling sites of the North Sea represent polyhaline water bodies (salinity 18‰ to 30‰) while the Baltic Sea sites are classified as mesohaline water bodies (salinity 5‰ to 18‰).

**Table 1:** Overview of the study area and sampling.

Bathing water site and ID	Coordinates [DMS]		Classification [EU-WFD]	No. of samples		Period
	°N	°E		Water	Sediment	
Dorum-Neufeld campsite Sandy/rocky beach DENI_PR_TK25_2217_02	53.7416	8.5139	Polyhaline Wadden Sea/transitional water; Weser estuary	15	14	May 2017–Aug 2018
Duhnen spa area Sandy beach DENI_PR_TK25_2117_04	53.8866	8.6376	Polyhaline Wadden Sea; Elbe estuary	15	14	May 2017–Aug 2018
Dyksterhusen oil rig (Dollart) Platform DENI_PR_TK25_2609_02	53.2940	7.2293	Transitional water; Ems estuary	15	15	May 2017–July 2018
Karlshagen main beach Sandy beach No. 703	54.7175	13.5053	Mesohaline open coastal waters	12	12	July 2017–Sep 2018
Lubmin sea-bridge Sandy beach No. 750	54.8172	13.3646	Mesohaline inner coastal waters	12	12	July 2017–Sep 2018
Warnemünde main beach Sandy beach No. 236	54.1051	12.4338	Mesohaline open coastal waters	15	15	Jun 2017–Aug 2018
Wohlenberger Wiek campsite Sandy beach No. 227	53.5554	11.1723	Mesohaline inner coastal waters	13	13	July 2017–Aug 2018

DMS: Degrees/Minutes/Seconds.

EU-WFD: European Water Framework Directive.

The German North Sea coast is characterized by tidal mud flats and estuaries of the rivers Elbe, Ems, and Weser, which comprise transitional waters between fresh and salt water. The sampling areas are located in Dyksterhusen within the Ems estuary, Dorum within the Weser estuary, and Duhnen, a district of Cuxhaven, within the Elbe estuary. The sampling sites of the Baltic Sea in Karlshagen, Lubmin, Warnemünde, and Wohlenberger Wiek are located in the inner to open coastal waters.

All locations are designated as official bathing waters according to the European bathing water directive (EBWD) and are popular for recreational activities such as wading, bathing, and water sports.

### Sampling

The monthly sampling of water and sediment was carried out by the local health authorities between May 2017 and August 2018 in accordance with the EBWD 2006/7/EC. Water samples were taken immediately after high tide times according to DIN EN ISO 19458:2006-12. Depending on the water level, surface sediments were taken using sterile sample containers or with sterile sampling devices and decanted afterwards. Environmental conditions at the sampling time, including air and water temperature and conductivity, were monitored as well by local health authorities. Within 24 h, the samples were shipped directly to the laboratory of the Federal Institute of Hydrology in Koblenz and analyzed immediately. During shipment, the samples were chilled with cool packs between fridge and room temperatures adapted to the seasonal conditions at the sampling site.

### Microbiological Detection and Quantification of *Vibrio* spp.

To quantify *Vibrio* spp. *via* microbiological culture-based methods, membrane filtration and direct plating have been applied for both water and sediment samples. Volumes of 10 ml were taken in triplicate directly from a homogeneous water sample and membrane filtered using a 0.2-µm pore size Whatman^®^ mixed cellulose ester filter. For sediment samples, sediment, distilled water, and artificial seawater (ASW; Sigma-Aldrich) were mixed in equal proportions. To acquire the bacteria from the sediment, the sediment was washed for 30 min on a magnetic stirrer. After allowing the sediment to settle, 10-ml triplicates of the supernatant were membrane filtered. Depending on the water temperature at the sampling site and the expected colony counts, decadal dilution series were prepared with ASW prior to filtration. After filtration, filters were transferred onto CHROMagar™ *Vibrio* (Mast Group) and incubated for 18–24 h at 37°C to favor the growth of potentially human pathogenic *Vibrio* species. Presumptive and oxidase positive *Vibrio* spp. colonies (Oxidase test stripes, Oxoid) were counted and converted to colony-forming units (CFU)/100 ml of water or 100 g of sediment.

Colonies were sub-cultured on CHROMagar™ *Vibrio* and thiosulfate citrate bile sucrose agar (TCBS, Merck) for 18–24 h at 37°C. For further differentiation, sub-cultured colonies were picked and incubated overnight in buffered peptone water (BPW supplement with an additional 1.5% NaCl concentration, Merck) at 37°C, mixed with glycerol, and stored at −80°C. Further biochemical and molecular biological analyses to identify potential human pathogenic *Vibrio* spp. were done with colonies grown in BPW or on Lysogenic broth (LB, Sigma-Aldrich) agar.

### Species-Specific Identification of *Vibrio* spp. and Detection of Virulence-Associated Genes

Presumptive *Vibrio* spp. colonies isolated by culture-based methods described above were transferred to matrix-assisted laser desorption ionization-time of flight mass spectrometry (MALDI-TOF MS) analyses for further species-specific identification. MALDI-TOF MS was performed with a microflex^®^ LT system mass spectrometer (Bruker Daltonik, Bremen, Germany) following the manufacturer`s settings. The MALDI-TOF MS spectra were obtained by applying the direct transfer protocol of bacterial colonies supplied by the manufacturer. The species identification was performed by running the spectra of isolates against the commercial database BDAL (Bruker Daltonik) and additional reference spectra that were created for some *Vibrio* species according to the manufacturer`s instruction. The MALDI-TOF MS technique provides a reliable pre-screening method for the first identification of potentially human-pathogenic *V. cholerae*, *V. parahaemolyticus*, and *V. vulnificus*. Furthermore, this technique also enabled an overview of other *Vibrio* species, which were present at the sampling sites.

Identified *V. cholerae*, *V. parahaemolyticus*, and *V. vulnificus* isolates were again confirmed by a probe based real-time PCR using the highly specific and sensitive *Vibrio* Detection LyoKit (Biotecon Diagnostics, Potsdam, Germany). Simultaneously to the species-specific identification, the *Vibrio* Detection LyoKit identified the cholera toxin (CTX) of *V. cholerae* encoded by the ctx gene, the thermostable direct hemolysin (TDH), and the TDH-related hemolysin (TRH) of *V. parahaemolyticus* encoded by *tdh* and *trh1*/*trh2* genes, respectively, by melting curves.

Virulence-associated genes for *V. vulnificus* were separately identified by PCR because it was not included in the *Vibrio* Detection LyoKit. The virulence-correlated *vcg* gene variant and the 16S rRNA type B gene variant associated with clinical *V. vulnificus* strains as well as the *cap*/*wcv* gene associated with the capsular polysaccharide (CPS) expression, which is known as a major virulence factor, were detected in accordance to Han and Ge (2010).

For *V. cholerae*, an additional serogroup PCR, modified in accordance to Mantri et al. (2006), was performed to identify fragments of the *O1rfb* (O1 serogroup specific rfb) and *O139rfb* (O139 serogroup specific *rfb*) gene regions. Each PCR reaction contained a mixture of 2.5 µl of 10× PCR buffer, 1.0 mM MgCl_2_, 8 mM dNTPs, 2.0 µl of Taq DNA polymerase at 5U µl^−1^, 0.5 µM of both O1rfb primers, 0.125 µM of both O139rfb primers, distilled water to a final volume of 25 µl, and 2.0 µl of DNA template (10 to 50 ng of DNA). O1rfb and O139rfb fragments were amplified as follows: initial denaturation at 94°C for 2 min, followed by 30 cycles consisting of 94°C for 30 s, 59°C for 30 s, and 72°C for 30 s, and a final extension at 72°C for 5 min.

As positive controls for the species-specific identification, the following strains were used: *V. cholerae* DSM101014, *V. parahaemolyticus* DSM101031, and *V. vulnificus* DSM10143. As positive controls for the detection of virulence-associated genes, the *V. cholerae* strains VN-0156 (*ctx*), VN-0147 (*O1rfb*), and VN-0150 (*O139rfb*); the *V. parahaemolyticus* strains VN-0088 (*tdh*) and RIMD 2210633 (*trh*); and the *V. vulnificus* strains ATCC 33815 (*vcg*, 16S rRNA type B variant, *cap*/*wcv*) were used.

### Quantification of *V. cholerae*, *V. parahaemolyticus*, and *V. vulnificus* by Real-Time PCR

To quantify *V. cholerae*, *V. parahaemolyticus*, and *V. vulnificus* in water and sediment samples, a quantitative real-time PCR using the *Vibrio* Detection LyoKit (Biotecon Diagnostics, Potsdam, Germany) was applied according to manufacturer’s instruction. For water samples, duplicates of 100 or 150 ml (depending on particle content) were membrane filtered using a 0.2-µm pore size Whatman^®^ mixed cellulose ester filter. The sediment samples were mixed in the same ratio with distilled water and ASW. Afterwards, the mixture was stirred on a magnetic stirrer for 30 min to detach the bacteria from the sediment. After stirring, duplicates of 50 ml were membrane filtered. DNA was extracted from the filters using the DNeasy PowerWater^®^ Kit (Qiagen, Hilden, Germany). The three target *Vibrio* species in each sample were quantified by means of standard curves using genomic DNA of *V. cholerae* DSM101014, *V. parahaemolyticus* DSM101031, and *V. vulnificus* DSM10143, which were also used as positive controls in each PCR run. Results were expressed as gene copy numbers of *V. cholerae*, *V. parahaemolyticus*, and *V. vulnificus* in 100 ml of water or 100 g of sediment and log10(copies+1)-transformed.

### Antibiotic Resistance Test

Sixteen antimicrobial agents (**Table 4**), commonly used in human medicine, were tested using the disk diffusion method to determine microbial susceptibility. Antimicrobial agents were applied with the following concentrations: beta-lactam antibiotics (amp-10, sam-20, atm-30, cef-30, fep-30, caz-30, cxm-30, mem-10, oxa-5, and pen-10), quinolone (cip-5, lvx-5, and nor-10), tetracycline (dox-30 and tet-30), and trimethoprim-sulfamethoxazole (ts-25). The disks were incubated on Mueller-Hinton agar (MHA, Carl Roth) plates inoculated with *Vibrio* isolates at 35°C for 16–18 h. The results were interpreted according to the Clinical and Laboratory Standards Institute (CLSI) guidelines from 2018 based on the diameter of the inhibition zone. Accordingly, the strains were defined as susceptible, intermediate resistant, and resistant.

### Statistical Analyses

Abundances of *Vibrio* spp. assessed by culture-based methods in water and sediment samples were based on triplicates. Species-specific identification, virulence gene detection, and the quantification *via* real-time PCR were done in duplicate. Results were expressed as median values in CFU/100 ml or g and copies/100 ml or g, respectively, and log-transformed (log10CFU+1/100 ml or g, log10copies+1/100 ml or g). For all statistical calculations, the open-source program R version 3.5.3 (R core team 2019) for statistical computing was used. Differences in *Vibrio* abundances between sampling sites, seasons, and sample matrices were analyzed using a multifactorial ANOVA in combination with the *post-hoc* test. Differences were considered significant when the *p*-value was ≤ 0.05.

## Results

### Spatial Distribution of Potentially Human Pathogenic *Vibrio* spp.

During monitoring study, the three potentially human pathogenic *Vibrio* species *V. cholerae*, *V. parahaemolyticus*, and *V. vulnificus* were detected at all seven recreational bathing sites on the German coast between 2017 and 2018. In total, 255 isolates were collected at the North Sea and 271 at the Baltic Sea. As confirmed by MALDI-TOF MS and species-specific gene targets using PCR, 68% (*n* = 173) of the North Sea and 60% (*n* = 163) of the Baltic Sea isolates were identified as one of the target species *V. cholerae*, *V. parahaemolyticus*, and *V. vulnificus.*

Within the group of target species, the North Sea isolates included 3% (*n* = 7) non-O1/non-O139 *V. cholerae*, 57% (*n* = 147) *V. parahaemolyticus*, and 7% (*n* = 19) *V. vulnificus*. The Baltic Sea isolates included 10% (*n* = 27) non-O1/non-O139 *V. cholerae*, 20% (*n* = 51) *V. parahaemolyticus*, and 31% (*n* = 85) *V. vulnificus* (**Table 2**). Accordingly, *V. parahaemolyticus* was the most frequent *Vibrio* target species in the North Sea samples, while *V. vulnificus* was the most frequent *Vibrio* target species in the Baltic Sea samples. Exclusively for the sampling sites Warnemünde main beach and Wohlenberger Wiek campsite at the Baltic Sea, it was shown by MALDI-TOF MS that the most frequently collected *Vibrio* spp. isolates belonged to the group of “other *Vibrio* species”.

**Table 2:** Overview of the collected mesophilic *Vibrio* spp. isolates with focus on *V. cholerae*, *V. parahaemolyticus*, and *V. vulnificus*.

Sampling area	Bathing water site	*V. cholerae* non-O1/non-O139	*V. parahaemolyticus*	*V. vulnificus*	Other *Vibrio* spp.
North Sea	Total number	3% (*n* = 7)	57% (*n* = 147)	7% (*n* = 19)	33% (*n* = 82)
	Dorum-Neufeld campsite	3% (*n* = 2)	48% (*n* = 30)	3% (*n* = 2)	45% (*n* = 30)
	Duhnen spa area	0% (*n* = 0)	51% (*n* = 31)	3% (*n* = 2)	46% (*n* = 28)
	Dyksterhusen oil rig (Dollart)	4% (*n* = 5)	65% (*n* = 86)	12% (*n* = 15)	20% (*n* = 24)
Baltic Sea	Total number	10% (*n* = 27)	20% (*n* = 51)	31% (*n* = 85)	39% (*n* = 108)
	Karlshagen main beach	28% (*n* = 17)	0% (*n* = 0)	56% (*n* = 34)	16% (*n* = 10)
	Lubmin sea-bridge	19% (*n* = 9)	2% (*n* = 1)	52% (*n* = 25)	27% (*n* = 13)
	Warnemünde main beach	0% (*n* = 0)	35% (*n* = 27)	7% (*n* = 6)	58% (*n* = 50)
	Wohlenberger Wiek campsite	1% (*n* = 1)	29% (*n* = 23)	25% (*n* = 20)	25% (*n* = 35)

The other collected *Vibrio* spp. isolates were identified as *V. aesturianus*, *V. alginolyticus*, *V. diazotrophicus*, *V. fluvialis*, *V. metschnikovii*, *V. mimicus*, and *V. navarrensis*. Within this group, *V. fluvialis* was the most frequent species at Warnemünde main beach (*n* = 24, 48%). *V. diazotrophicus* (*n* = 12, 34%), and *V. fluvialis* (*n* = 12, 34%) were the most frequent other *Vibrio* species at the Wohlenberger Wiek campsite. *V. alginolyticus* was the most frequent other *Vibrio* species at Dorum-Neufeld campsite and Duhnen spa area (*n* = 28, 3% and *n* = 25, 89%, respectively).

Regarding the sampling sites Lubmin sea-bridge and Karlshagen main beach (**Table 2**), an increased number of *V. cholerae* isolates in relation to the other sampling sites were detected (*n* = 9, 19% and *n* = 17, 28%, respectively).

### Seasonal Distribution of Potentially Human Pathogenic *Vibrio* spp.

**Figure 1:**
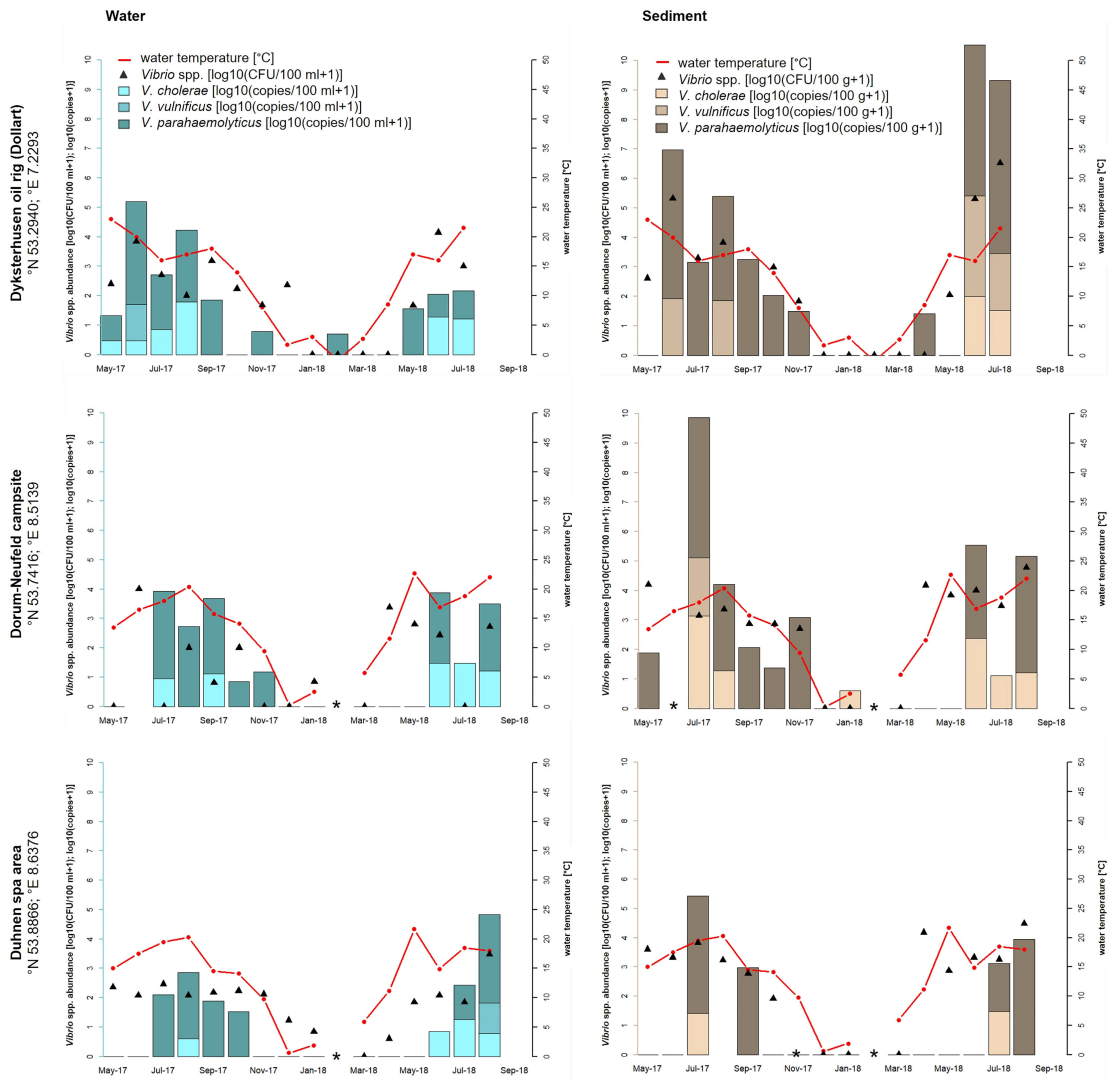
Seasonal distribution of *Vibrio* spp. including the proportion of *V. cholerae*, *V. parahaemolyticus*, and *V. vulnificus* from water and sediment samples at the three sampling sites of the North Sea: Dyksterhusen oil rig (Dollart), Dorum-Neufeld campsite, and Duhnen spa area. The red line presents the seawater temperature, the black triangles (median value of a triplicate) show the *Vibrio* spp. abundance of all detected *Vibrio* species with culture-based methods in log10 CFU+1 per 100 ml of water or 100 g of sediment, and the bars show the quantitative real-time PCR results in log10 copies+1 reflecting the proportion of *V. cholerae*, *V. parahaemolyticus*, and *V. vulnificus* in the water (blue bars) and sediment (brown bars) samples. *Samples were not taken.

**Figure 2:**
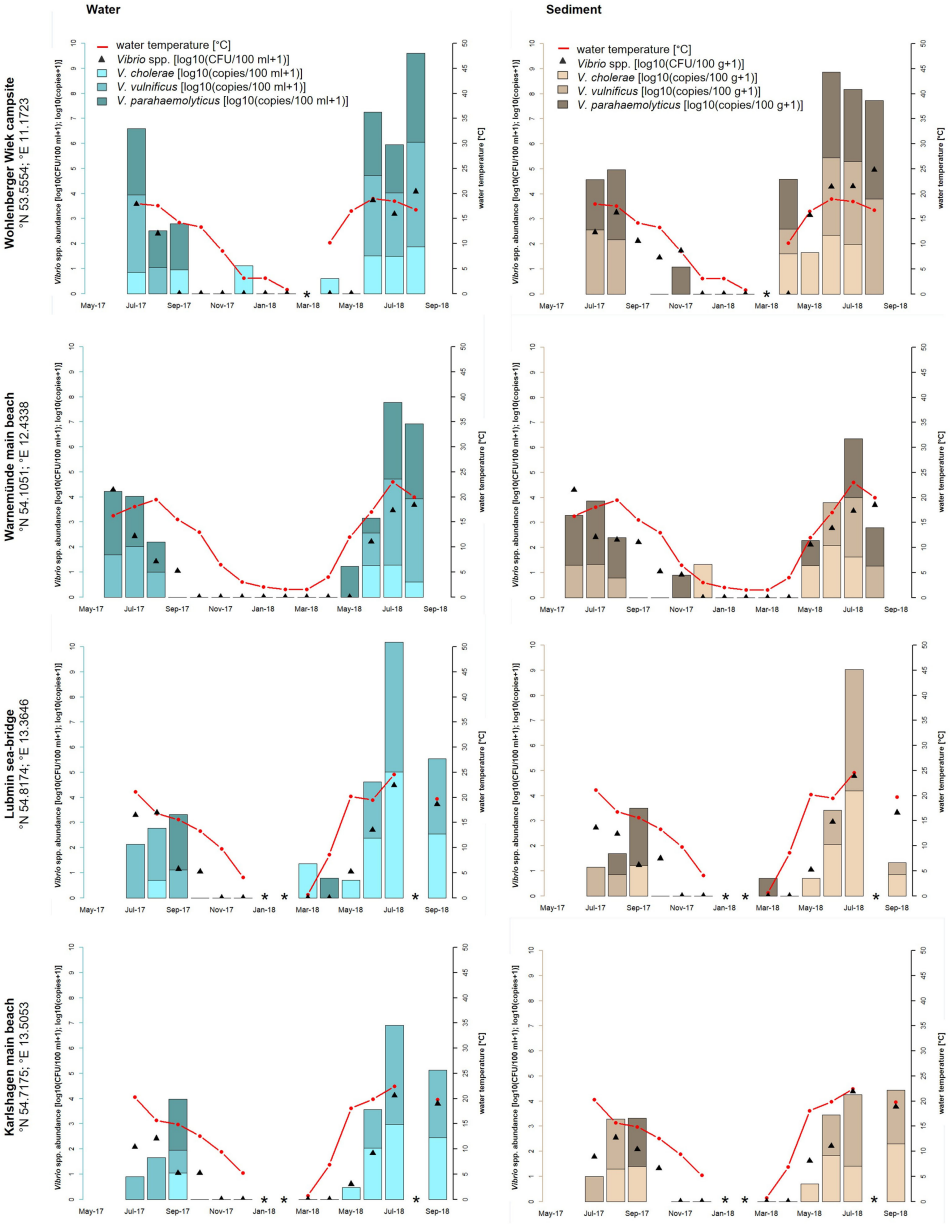
Seasonal distribution of *Vibrio* spp. including the proportion of *V. cholerae*, *V. parahaemolyticus*, and *V. vulnificus* from water and sediment samples at the four sampling sites of the Baltic Sea: Wohlenberger Wiek campsite, Warnemünde main beach, Lubmin sea-bridge, and Karlshagen main beach. The red line presents the seawater temperature, the black triangles (median value of a triplicate) show the *Vibrio* spp. abundance of all detected *Vibrio* species with culture-based methods in log10 CFU+1 per 100 ml of water or 100 g of sediment, and the bars show the quantitative real-time PCR results in log10 copies+1 reflecting the proportion of *V. cholerae*, *V. parahaemolyticus*, and *V. vulnificus* in the water (blue bars) and sediment (brown bars) samples. *Samples were not taken.

A strong seasonal distribution of *Vibrio* spp. including *V. cholerae*, *V. parahaemolyticus*, and *V. vulnificus* (ANOVA, *p* < 0.001) was observed in this study (see **Figures 1**, **2**). Water temperatures (red line) recorded at the North Sea sampling sites ranged between −1.0°C (Dyksterhusen oil rig, February 2018) and 22.7°C (Dorum-Neufeld campsite, May 2018). Highest temperatures were measured at the Baltic Sea. There, temperatures ranged between 0.6°C and 24.6°C (Lubmin sea bridge, February 2018 and July 2018, respectively). With water temperatures rising above 11.2°C (April 2018) at the North Sea sampling sites and 12°C (May 2018) at the Baltic Sea sampling sites, *Vibrio* abundances rose explosively, as shown by culture-based methods (black triangles) as well as quantitative real-time PCR (bars).

High-water temperatures occur primarily during the whole study time between April/May to August/September. At the North Sea, culture-based analyses revealed highest *Vibrio* spp. abundances in June 2018 at Dyksterhusen oil rig in water (4.1 log10 CFU+1/100 ml) and sediment (6.5 log10 CFU+1/100 g). At the Baltic Sea, the highest abundances were found in August 2018 at the Wohlenberger Wiek campsite in water (4.1 log10 CFU+1/100 ml) and sediment (5.0 log10 CFU+1/100 g) as well as in July 2018 at Lubmin sea-bridge in water (4.5 log10 CFU+1/100 ml) and sediment (4.8 log10 CFU+1/100 g). In general, *Vibrio* spp. abundances were up to three log higher in sediment than in water (ANOVA, *p* < 0.05) and allowed a mesophilic *Vibrio* spp. detection until November 2017. However, mesophilic *Vibrio* spp. were recovered from water samples sporadically during the winter period until January 2018. In general, *Vibrio* spp. were not detected *via* culture-based methods in the North Sea between February and March 2018 with water temperatures between −1.0 and 5.9°C and in the Baltic Sea between December 2017 and April 2018 with water temperatures between 0.6 and 8.6°C.

As opposed to the culture-based methods, quantitative real-time PCR allowed a species-specific quantification of the target species *V. cholerae*, *V. parahaemolyticus*, and *V. vulnificus.* Species-specific abundances of the target species and their relative proportions are shown as stacked bars. In the North Sea samples, *V. cholerae* was detected only during summer between May and September when water temperature rose above 15°C (except for the Dorum-Neufeld campsite in January 2018). During this time, *V. cholerae* reached concentrations up to 1.8 log10 copies+1/100 ml in water (Dyksterhusen oil rig, August 2017) and 3.1 log10 copies+1/100 g in sediment (Dorum-Neufeld campsite, July 2017). In total, *V. cholerae* was detected in 24% (*n* = 21 of 88) of the North Sea samples. *V. parahaemolyticus* was shown to be the most abundant species in the North Sea, as it was detected in half (*n* = 44 of 88) of the samples between April and November (except for the Dyksterhusen oil rig in February 2018). Concentrations of *V. parahaemolyticus* reached up to 3.5 log10 copies+1/100 ml in water (Dyksterhusen oil rig, June 2017) and 5.9 log10 copies+1/100 g in sediment (Dyksterhusen oil rig, July 2018). *V. vulnificus* was detected in 8% (*n* = 7 of 88) of the North Sea samples and was therefore the least detected *Vibrio* species of the three focused species. A detection of *V. vulnificus* was only possible between June and August when the water temperature was above 15°C. Concentrations ranged up to 1.4 log10 copies+1/100 ml (Dyksterhusen oil rig, June 2017) in water and 3.4 log10 copies+1/100 g in sediment (Dyksterhusen oil rig, June 2018).

In the Baltic Sea, *V. cholerae* and *V. parahaemolyticus* were mainly detected during the summer period between May and September when the water temperature rose above 15°C. During this time, *V. cholerae* reached concentrations up to 5.0 log10 copies+1/100 ml in water and 4.3 log10 copies+1/100 g in sediment (Lubmin sea-bridge, July 2018). *V. parahaemolyticus* concentrations reached up to 3.6 log10 copies+1/100 ml in water and 3.9 log10 copies+1/100 g in sediment (Wohlenberger Wiek campsite, July 2018). From October to April with temperatures between 0 and 15°C, *V. parahaemolyticus* occurred sporadically except at Karlshagen main beach. In total, *V. cholerae* was detected in 39% (*n* = 40 of 104) and *V. parahaemolyticus* was detected in 33% (*n* = 34 of 104) of the Baltic Sea samples. *V. vulnificus* was found in 43% (*n* = 45 of 104) of the Baltic Sea samples and was therefore the most frequent *Vibrio* species of the three focused species, although a detection was only possible between June and September when the water temperature exceeds 15°C (except Wohlenberger Wiek campsite, April 2018). Concentrations ranged up to 5.2 log10 copies+1/100 ml in water and 4.8 log10 copies+1/100 g in sediment (Lubmin sea-bridge, July 2018).

### Effect of Salinity on the Distribution of *Vibrio* spp.

**Figure 3:**
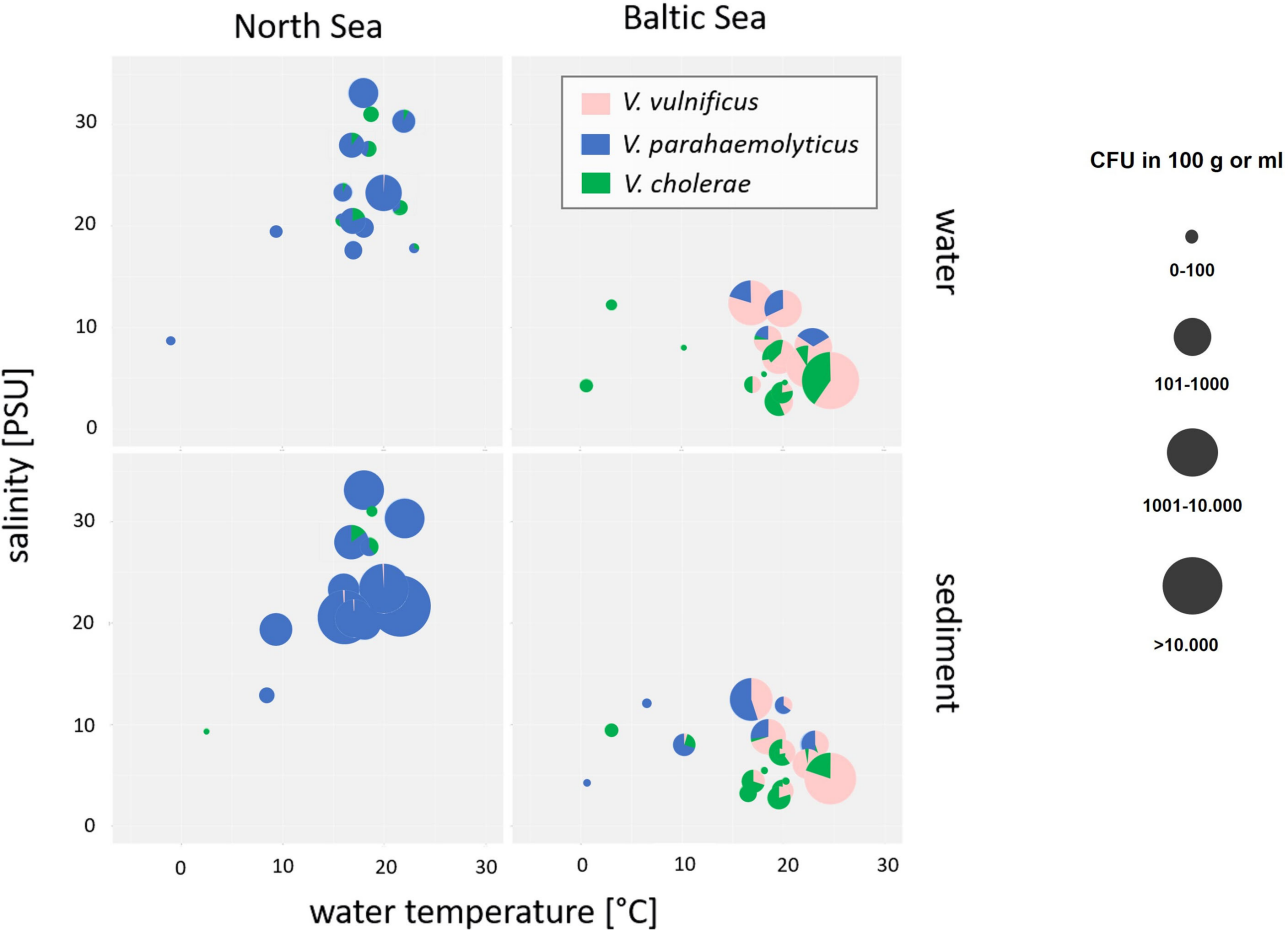
Bubble pie chart showing abundances and relative proportions of *V. cholerae*, *V. parahaemolyticus*, and *V. vulnificus* in dependence of salinity and water temperature in water and sediment samples of North Sea and Baltic Sea samples. *V. cholerae* is shown in green, *V. parahaemolyticus* is shown in blue, and *V. vulnificus* is shown in pink. The bubble size represents the concentration in log10 CFU+1/100 ml of water or 100 g of sediment.

Next to the species-specific response to the water temperature, the salinity represents the main determinant to influence the *Vibrio* community. Abundances and relative proportions of *V. cholerae*, *V. parahaemolyticus*, and *V. vulnificus* in dependence of salinity and temperature are shown in **Figure 3**.

Salinities at the North Sea sampling sites ranged between 18‰ and 33‰ while salinities at the Baltic Sea sampling sites were considerably lower and ranged between 2‰ and 13‰ by water temperatures at 20°C. *V. parahaemolyticus* was found in environments with salinities from 7‰ to 33‰ and might be adapted to high salinity concentrations of the North Sea (ANOVA, *p* < 0.001). In contrast, *V. vulnificus* was found in environmental samples with salinities from 3‰ to 13‰ and seems to prefer the moderate salinity concentrations of the Baltic Sea (ANOVA, *p* < 0.001). The data correspond to other publications (Martinez-Urtaza et al., 2010; Froelich and Oliver, 2013; Froelich et al., 2015). In rare cases, *V. vulnificus* was detectable in North Sea samples by salt concentrations between 21‰ and 33‰, whereas *V. parahaemolyticus* was detectable by a salt concentration of 5‰. Non-O1/non-O139 *V. cholerae* occupy both habitats of moderate and high salt concentrations and could be found in North Sea as well as in Baltic Sea samples, whereas low salt concentrations between 3‰ and 7‰ were optimal concentrations to detect *V. cholerae* in water and sediment samples even though sporadic detections were obtained up to 31‰ salinity. However, *V. cholerae* detections were associated with estuarine samples.

### Virulence-Associated Genes

Environmental isolates of the target species were analyzed by PCR for the presence of selected virulence-associated genes (**Table 3**). TDH-related hemolysin gene variants *trh1* and *thr2* were found in *V. parahaemolyticus* isolates from both North Sea and Baltic Sea sites. The thermostable direct hemolysin gene *tdh* was not detected.

**Table 3:** Overview of virulence-associated genes among the collected *V. cholerae*, *V. parahaemolyticus*, and *V. vulnificus isolates*.

Area	*V. cholerae* non-O1/non-O139	*V. parahaemolyticus*			*V. vulnificus*		
	*ctx*	*trh1*	*trh2*	*tdh*	*cap/wcv*	*vcgC*	16S rRNA-type B
North Sea	0% (*n* = 0 of 7)	7% (*n* = 8 of 119)	18% (*n* = 21 of 119)	0% (*n* = 0 of 119)	28% (*n* = 5 of 18)	11% (*n* = 2 of 18)	17% (*n* = 3 of 18)
Baltic Sea	0% (*n* = 0 of 27)	6% (*n* = 3 of 53)	9% (*n* = 5 of 53)	0% (*n* = 0 of 53)	18% (*n* = 9 of 85)	0% (*n* = 0 of 85)	55% (*n* = 27 of 85)

ctx, cholera toxin gene (ctx); trh1 and trh2, TDH-related hemolysin gene variant 1 and 2; tdh, thermostable direct hemolysin gene; vcgC, virulence correlated gene; 16S rRNA type B, gene variant for clinical V. vulnificus; cap/wcv, gene involved in capsular polysaccharide expression.

Virulence-associated genes of *V. vulnificus* including the *vcgC* gene variant, the 16S rRNA type B gene variant, as well as the gene *cap/wcv* were also found. The presence of the investigated virulence genes differed in the *V. vulnificus* isolates obtained from the North Sea and Baltic Sea. The *cap/wcv* gene was detected in 28% (*n* = 5 of 18) and the 16S rRNA type B gene variant in 17% (*n* = 3 of 18) of the *V. vulnificus* isolates from the North Sea. In the *V. vulnificus* isolates from the Baltic Sea, the *cap/wcv* gene was detectable in 18% (*n* = 9 of 85) and the 16S rRNA type B gene variant was detectable in 55% (*n* = 27 of 85). The *vcgC* gene was only detectable in 11% (*n* = 2 of 18) of the *V. vulnificus* isolates collected from the North Sea areas. Furthermore, virulence-associated genes were only detected in *V. parahaemolyticus* and *V. vulnificus* isolates collected between April and September when an increase of *Vibrio* spp. was observed. All *V. cholerae* isolates were identified as non-O1/non-O139 serotypes without the cholera toxin gene *ctx*.

### Antibiotic Resistance

All of the examined non-O1/non-O139 *V. cholerae* isolates were susceptible to the majority of the antimicrobial agents, which were selected for this study (**Table 4**). Only 9% (*n* = 3) of the isolates showed an intermediate resistance to meropenem and a resistance to ampicillin. Intermediate resistance to cefazolin with 32% (*n* = 11) and resistances to cefazolin with 53% (*n* = 18), oxacillin with 100% (*n* = 34), penicillin with 100% (*n* = 34), and trimethoprim-sulfamethoxazole with 91% (*n* = 31) were observed. Therefore, *V. cholerae* isolates showed only resistance to the beta-lactam antibiotics (cefazolin, oxacillin, and penicillin) and to the trimethoprim-sulfamethoxazole combination.

**Table 4:** Overview of the efficacy of antimicrobial agents towards the collected *V. cholerae*, *V. parahaemolyticus*, and *V. vulnificus isolates*.

Antimicrobial agent ^a^	*V. cholerae* non-O1/non-O139 *n* = 34			*V. parahaemolyticus n* = 198			*V. vulnificus n* = 102		
	S (%)	I (%)	R (%)	S (%)	I (%)	R (%)	S (%)	I (%)	R (%)
Ampicilin and Sulbactam	100	0	0	100	0	0	99	0	1
Ampicillin	91	0	9	5	5	90	94	0	6
Aztreonam	100	0	0	99	1	0	97	3	0
Cefazolin	15	32	53	2	31	67	97	2	1
Cefepime	100	0	0	99	1	0	92	8	0
Ceftazidim	100	0	0	99	1	0	99	1	0
Cefuroxim	100	0	0	79	19	2	97	2	1
Ciproflaxacin	100	0	0	100	0	0	99	1	0
Meropenem	91	9	0	99	1	0	98	2	0
Oxacillin	0	0	100	0	0	100	0	0	100
Penicillin	0	0	100	0	0	100	0	0	100
Trimethoprim-sulfamethoxazole	9	0	91	46	14	40	66	15	19

S, susceptible; I, intermediate resistant; R, resistant.

a All non-O1/non-O139 V. cholerae, V. parahaemolyticus, and V. vulnificus isolates were susceptible to doxycycline, levofloxacin, norfloxacin, and tetracycline.

Regarding the 198 collected *V. parahaemolyticus* isolates, susceptibilities to the majority of the antimicrobial agents were observed similar to the non-O1/non-O139 *V. cholerae* isolates (**Table 4**). Seventy-nine percent (*n* = 158) of the isolates were also susceptible to cefuroxime and only 2% (*n* = 3) displayed a resistance. The trimethoprim- sulfamethoxazole combination showed susceptibilities to 46% (*n* = 90) and resistances to 40% (*n* = 80) of the isolates. Resistances to ampicillin with 90% (*n* = 179), cefazolin with 67% (*n* = 132), and oxacillin and penicillin with 100% (*n* = 198) were determined. Therefore, *V. parahaemolyticus* isolates showed resistance only to the beta-lactam antibiotics ampicillin, cefazolin, oxacillin, and penicillin, as well to trimethoprim-sulfamethoxazole.

The majority of the 102 collected *V. vulnificus* isolates were susceptible to the antimicrobial agents, which were selected for this study (**Table 4**) except for oxacillin, penicillin, and trimethoprim-sulfamethoxazole. Whereas all *V. vulnificus* isolates displayed resistance to the beta-lactam antibiotics oxacillin and penicillin, 19% (*n* = 19) of the isolates showed a resistance and 66% (*n* = 68) are susceptible to trimethoprim-sulfamethoxazole.

## Discussion

### Spatial and Seasonal Distribution of Potentially Human Pathogenic *Vibrio* spp.

Potentially human pathogenic *V. cholerae*, *V. parahaemolyticus*, and *V. vulnificus* were detected at all seven recreational bathing areas along the German North Sea and Baltic Sea coasts. During the sampling time from May 2017 to September 2018, a heat wave in Europe starting in April 2018 holding on to August 2018 was included in the monitoring study. During the last heat wave in Europe in summer 2014, Baker-Austin et al. (2016) reported increased numbers of infections with 89 cases of vibrioses in Sweden and Finland (Baker-Austin et al., 2016). High numbers of *Vibrio* infections also occurred during the heat wave in 2018. The LAGuS reported three deaths and 17 *Vibrio* infections, which were related to direct water contact during bathing in the Baltic Sea in Mecklenburg-Western Pomerania (LAGuS, 2018). Metelmann et al. (2020) showed a proportional significant correlation between rising water temperatures on the coasts of Mecklenburg-Western Pomerania and infections with *V. vulnificus* including severe cases of sepsis during the bathing season. In total, three deaths and 10 of the 17 *Vibrio* infections in summer 2018 could be attributed to a septicemia with *V. vulnificus*. These numbers are the highest ever recorded in a bathing season in Germany. The highest water temperature of the Baltic Sea, which was measured in this study, reached 24.6°C at Lubmin sea-bridge in July 2018. The local *Vibrio* spp. detection rates *via* culture-based methods, 4.5 log10 CFU+1/100 ml of water and 4.8 log10 CFU+1/100 g of sediment, were also the highest in this study. Concentrations of *V. vulnificus*, measured *via* real-time PCR, reached in that time at this sampling site 5.2 log10 copies+1/100 ml of water and 4.8 log10 copies+1/100 g of sediment. Comparable high detection numbers were achieved for *Vibrio* spp. at the sampling site Wohlenberger Wiek campsite with 4.1 log10 CFU+1/100 ml of water and 5.0 log10 CFU+1/100 g of sediment in August 2018. Regarding the quantitative real-time PCR results, concentrations of *V. vulnificus* reached 4.0 log10 copies+1/100 ml of water and 100 g of sediment. During the monitoring, *V. vulnificus* was the dominant *Vibrio* species at all four sampling sites along the Baltic Sea coast during the bathing season between June and September 2018 (43%, *n* = 45 of 104) (see **Figure 2**). These findings corroborate the high numbers of *V. vulnificus* sepsis cases reported by Metelmann et al. (2020). However, not only wound infections with *V. vulnificus* increase significantly, infections with non-O1/non-O139 *V. cholerae* were also recorded more frequently (Brehm et al., 2021). According to additional investigations, besides the reports published by the LAGuS, a total of 47 cases of infection due to non-cholera-*Vibrio* infections were confirmed by Brehm et al. (2021) on the German North Sea and Baltic Sea coasts in 2018. Thereby, *V. vulnificus*, with 41%, was the most frequent pathogenic *Vibrio* species, whereas non-O1/non-O139 *V. cholerae*, with 29%, was by far the second most frequent pathogenic *Vibrio* species (Brehm et al., 2021). These findings can also be confirmed in this study detecting non-O1/non-O139 *V. cholerae* during the monitoring in 39% (*n* = 40 of 104) of the Baltic Sea samples and in 24% (*n* = 21 of 88) of the North Sea samples *via* quantitative real-time PCR. The highest detection numbers of non-O1/non-O139 *V. cholerae* were found at the Baltic Sea sampling site Lubmin sea-bridge in July 2018 with 5.0 log10 copies+1/100 ml of water and 4.3 log10 copies+1/100 g of sediment, respectively.

Increased *Vibrio* spp. detection numbers in combination with high water temperatures were also measured at the three sampling sites on the North Sea coast during this study. The highest temperature was obtained in May 2018 with 22.7°C at the Dorum-Neufeld campsite. Additionally, the highest *Vibrio* spp. concentrations were reached in June/July 2018 with 4.1 log10 CFU+1/100 ml of water and 6.5 log10 CFU+1/100 g of sediment at Dyksterhusen oil rig. Comparing these results to a previous monitoring study in 2010 to 2011 from Boer et al. (2013), an increase of *Vibrio* spp. of 1.5 log for both water and sediment samples were observed in this study at the North Sea. However, wound infections or septicemia in connection with bathing were not reported to the local health authorities in Lower Saxony at the German North Sea in 2018. The low frequency of *V. vulnificus* detected with 8% (*n* = 7 of 88) *via* quantitative real-time PCR in this study coincides with the lack of infection cases. The highest *V. vulnificus* concentrations with 1.4 log10 copies+1/100 ml of water and 3.4 log10 copies+1/100 g of sediment were detected at the sampling site Dyksterhusen oil rig (see **Figure 1**), comparable to Boer et al. (2013). These detection numbers are considerably lower than the concentration of *V. vulnificus* at the Baltic Sea. From 2003 to 2020, only 2% of the reported non-cholera-*Vibrio* infections by Brehm et al. (2021) could be attributed to the North Sea while 98% could be attributed to the Baltic Sea. Nevertheless, increased detection numbers of non-O1/non-O139 *V. cholerae* are observed with 24% (*n* = 21 of 88) by real-time PCR in contrast to the study by Boer et al. (2013) with a detection number of only 3% using culture-dependent methods. Detection numbers were also 1 log higher in water with 1.8 log10 copies+1/100 ml and 2.4 log higher in sediment with 3.1 log10 copies+1/100 g. High frequencies of *V. parahaemolyticus* with 50% (*n* = 44 of 88) at the North Sea and 33% (*n* = 34 of 104) at the Baltic Sea including very high detection numbers up to 5.9 log10 copies+1/100 g of sediment (Dyksterhusen oil rig, July 2018) were found *via* quantitative real-time PCR, comparable to the study of Boer et al. (2013). However, extraintestinal infections with *V. parahaemolyticus* at the German North Sea and Baltic Sea are rare (8%) and play a subordinate role (Brehm et al., 2021).

In this study, an increase in *Vibrio* numbers was observed above a seawater temperature of ≥10°C, reaching the highest detection numbers at 20 and 25°C. This observation correlated exactly with other studies, for example from Martinez-Urtaza et al. (2010); Baker-Austin et al. (2013), and Baker-Austin et al. (2017). Since 2010, the European land surface temperature has risen by 1.2°C (NOAA, 2010–2020). Especially in the year 2018, the water surface temperature was 2.0°C above the average temperature of the 20th century at the North Sea and 2.8°C at the Baltic Sea (BSH, 2020). The effect of rising water surface temperatures leads to rapidly increasing detection numbers of *Vibrio* spp. in spring, reaching the highest detection numbers in the early summer period, holding on to late in fall and dropping down in winter. Referring to the sampling points at the North Sea (**Figure 1**), *Vibrio* spp. could be detected until January 2018. Temperatures of −1.0°C were measured in February 2018 and a detection *via* culture-based methods was not possible until March 2018 at temperatures of 5.9°C. An increase in *Vibrio* numbers could be observed starting in April 2018 at 11.2°C, reaching the highest temperatures of 22.7°C in May 2018 and the highest detection numbers in June/July 2018. Persistently high detection numbers of *Vibrio* spp. could be detected between April/May and October/November. A similar observation could be revealed at the Baltic Sea (**Figure 2**). From December 2017 to April 2018, a detection *via* culture-based methods at temperatures between 0.6 and 8.6°C was not possible. A possible hypothesis therefore can be the VBNC-State. However, the lack of detection of species-specific gene targets for *V. cholerae*, *V. parahaemolyticus*, and *V. vulnificus* using quantitative real-time PCR (**Figures 1**, **2**), which should detect bacteria in VBNC-state, suggest that these *Vibrio* species are not present in the samples. A rapid increase of *Vibrio* spp. could be observed starting in May 2018 at 12°C, reaching the highest temperatures at 24.6°C in July 2018 and the highest detection numbers in July/August 2018. Cantet et al. (2013) confirmed these observations in a monitoring study at coastal lagoons of the French Mediterranean. In this study, persistently high detection numbers of *Vibrio* spp. were detected between April/May to September/October. In order to examine the seasonal infection risk period along the European coasts from May to October, reported by the CDC from 2017, this study indicates an even slightly longer infection risk period from April until November. In sediments, *Vibrio* spp. concentrations were up to three log higher than in water samples, suggesting that sediments play an important role for *Vibrio* spp. persistence in the aquatic environment. These findings were also confirmed in a previous study by Boer et al. (2013). The assumption of a circulation of *Vibrio* spp. between the seawater and the sediment is likely. In particular, during wave action, resuspension of *Vibrio* spp. from the sediment could be a possible scenario. However, such dynamics of *Vibrio* spp. have not been seriously discussed. Nevertheless, contact with sediment represents an increased risk potential of infections compared to direct water contact. Therefore, infections caused by wading in the North Sea and Baltic Sea can also play an important role in addition to bathing.

### Effect of Salinity on the Distribution of *Vibrio* spp.

Whereas the temperature was the most important parameter to detect *Vibrio* spp. at the sampling areas, the salinity was the most important determinant that affects the species-specific *Vibrio* spp. community (**Figure 3**). In particular, the three studied *Vibrio* species differ in their optimal salinity requirements and tolerances. Whereas *V. cholerae* is adapted to low salinity concentrations (0 to 8‰) (Huq et al., 2005), the optimal salinity for *V. parahaemolyticus* ranged from 10‰ to 34‰ and that for *V. vulnificus* ranged from 5‰ to 25‰. *Vibrio* spp. infections including infections with *V. vulnificus* are most common when the water temperature is ≥15°C and the salinity is between 2‰ and 25‰ (Randa et al., 2004; Vezzulli et al., 2013; Froelich et al., 2015; Baker-Austin et al., 2018; Froelich and Daines, 2020; Metelmann et al., 2020). In this study, the sampling areas at the Baltic Sea (**Table 1**) include exactly these specific salinity conditions with a mesohaline characteristic of salinities between 5‰ and 13‰. In contrast, the sampling areas at the North Sea do not include the entire range of this specific salinity conditions with a polyhaline characteristic of salinities between 18‰ and 33‰. Regarding the species-specific salinities for optimal growth conditions, *V. parahaemolyticus* was found in this study as the dominant *Vibrio* species around the North Sea with salt concentrations between 7‰ and 33‰ (**Figure 3**) confirming the findings from Boer et al. (2013). In contrast, *V. vulnificus* was found to be the dominant *Vibrio* species with salt concentrations between 3‰ and 13‰ at the Baltic Sea (**Figure 3**) mainly at the sampling sites Lubmin sea-bridge and Karlshagen main beach (**Table 2**). In addition, *V. cholerae* was also associated in this study with both sampling sites at the Baltic Sea (**Table 2**) with low salt concentrations between 3‰ and 7‰ (**Figure 3**). With its moderate salt concentration (3‰–18‰) and the possibility of a rapid rise in water temperatures due to geographic conditions, the Baltic Sea is an ideal aquatic ecosystem for the spread of *V. vulnificus* and *V. cholerae* (Bier et al., 2013; Huehn et al., 2014; Bier et al., 2015; Metelmann et al., 2020). However, highly pathogenic *V. vulnificus* strains were also reported to occur in the North Sea especially in the areas of the Weser and Ems estuaries by salt concentrations between 15‰ and 25‰ (Boer et al., 2013; Huehn et al., 2014; Bier et al., 2015; Metelmann et al., 2020). In this study, a sporadic detection of *V. vulnificus* and *V. cholerae* could also be observed at the Weser, Elbe, and Ems estuary sampling sites up to a salinity of 33‰ (**Table 2**), confirming the occurrence of both of these potentially pathogenic *Vibrio* species at North Sea areas. These results suggest that *V. vulnificus* and *V. cholerae* originating from estuaries in regions with lower salinities can persist in aquatic habitats with polyhaline characteristics.

### Detection of Virulence-Associated Genes

Previous studies have shown that *Vibrio* isolates from marine habitats, seafood, and clinical sources show a high genetic variance suggesting a large number of different pathogenicity mechanisms (Huehn et al., 2014). Nevertheless, potentially human pathogenic *Vibrio* have been identified in this study that harbor genes associated with clinical *Vibrio* strains.

Pathogenic *V. vulnificus* are known to possess a special *vcgC* gene variant, a 16S rRNA-type B gene variant, and the *cap/wcv* gene, which is directly involved in the capsular polysaccharide expression (Han and Ge, 2010). All three virulence factors were detected in the *V. vulnificus* isolates collected at the North Sea sampling areas (**Table 3**). The *vcgC* gene variant lacked all *V. vulnificus* isolates from the Baltic Sea sampling areas, but the 16S rRNA-type B gene variant with 55% (*n* = 27 of 85) was more observed than in North Sea isolates with 17% (*n* = 3 of 18), confirming a previous study from Bier et al. (2015). The presence of all three virulence factors in one isolate was not found in this study. However, 45% (*n* = 46 of 103) of the *V. vulnificus* isolates from both sampling areas possessed at least one virulence factor. The major virulence factor *cap/wcv* was present in 28% (*n* = 5 of 18) of the *V. vulnificus* isolates from the North Sea and in 18% (*n* = 9 of 85) of the Baltic Sea isolates. Therefore, potentially human pathogenic *V. vulnificus* occurs in the *Vibrio* communities of the German North Sea and Baltic Sea coasts and could cause infection cases as reported by Metelmann et al. (2020).

The pathogenicity of *V. parahaemolyticus* correlates with the presence of a thermostable direct hemolysin (TDH) and the TDH-related hemolysin (TRH) with up to 84% homologous variants TRH1 and TRH2 (Kishishita et al., 1992; Nishibuchi et al., 1992; Lee et al., 2002). Both *trh* gene variants were present in *V. parahaemolyticus* isolates at the sampling areas of the North Sea and Baltic Sea while *tdh* was not detected (**Table 3**). As already mentioned, extraintestinal infections caused by *V. parahaemolyticus* are rare (Brehm et al., 2021) but the haemolysins play an important role for gastrointestinal infections (Makino et al., 2003; Park et al., 2004). Nevertheless, the presence of the hemolysins is an accepted marker for the pathogenicity of *V. parahaemolyticus* strains (Ceccarelli et al., 2013) and illustrates that potentially human pathogenic strains are present at the German coasts.

All *V. cholerae* isolates were identified as non-O1/non-O139 serotypes without the cholera toxin (CTX). However, recent studies indicate that non-O1/non-O139 *V. cholerae* strains can cause extraintestinal infections by direct water contact next to cholera-like diarrheal diseases (Restrepo et al., 2006; Schirmeister et al., 2014; Deshayes et al., 2015), even though they do not produce the main cholera virulence factors. It is suggested that the virulence mechanisms of non-O1/non-O139 *V. cholerae* strains are more variable than those of the O1/O139 serogroups, but these mechanisms have not been fully characterized yet (Schirmeister et al., 2014; Baker-Austin et al., 2018).

### Detection of Antibiotic Resistant *Vibrio* spp.

All collected non-O1/non-O139 *V. cholerae*, *V. parahaemolyticus*, and *V. vulnificus* isolates of this study were susceptible to most of the tested antibiotics and no multidrug resistance was observed (**Table 4**). These results were also confirmed by a previous study from Bier et al., 2015, characterizing the antimicrobial resistance patterns in non-O1/non-O139 *V. cholerae* and *V. vulnificus* isolates from German coastal waters. Among all the collected non-O1/non-O139 *V. cholerae*, *V. parahaemolyticus*, and *V. vulnificus* isolates, only resistances to beta-lactam antibiotics (ampicillin, cefazolin, oxacillin, and penicillin) and trimethoprim-sulfamethoxazole were found. In a previous study from Huehn et al. (2014), isolates have already been detected with carbapenem-hydrolyzing beta-lactamases in German coastal waters. However, Bier et al. (2015) showed that non-O1/non-O139 *V. cholerae* and *V. vulnificus* have already increased resistances to aminoglycosides, aminopenicillins, streptomycin, and kanamycin (Bier et al., 2015). Due to the high genetic variability of *Vibrio* species, an increase in antibiotic resistance through the transmission of plasmid-encoded resistances and other mobile genetic elements is possible. However, an early antibiotic treatment is crucial to avoid septicemia, amputations, and a fatal course of the infection, if a wound infection by *Vibrio* species is suspected. A combined antibiosis of third-generation cephalosporin, tetracycline, and ciprofloxacin is recommended to ensure that there is no antibiotic resistance (CDC, 2017; Metelmann et al., 2020; Brehm et al., 2021).

## Conclusion

To sum up, a general increase in water temperatures affects an increase of *Vibrio* spp. concentrations, including potentially human pathogenic *Vibrio* species, on German coasts. Additionally, heat waves in spring and summer support an explosive spread of these bacteria. These facts are the main cause for an increasing risk of *Vibrio* infections by direct water contact with eyes, ears, skin, or wounds by bathing or wading at the German North Sea and Baltic Sea coasts. Besides water temperature, salinity was the most important determinant affecting the spatial distribution of the *Vibrio* spp. community. Therefore, *V. parahaemolyticus* was found to be the dominant *Vibrio* species in the North Sea with its polyhaline characteristics, while *V. vulnificus* was found to be the dominant *Vibrio* species in the Baltic Sea with its mesohaline characteristics. Combining this monitoring study and the educational work by Metelmann et al. (2020), a clear connection between the heat wave in 2018 and the increase of *V. vulnificus* infections could be seen at recreational bathing areas of the Baltic Sea. During the sampling time, multidrug-resistant non-O1/non-O139 *V. cholerae*, *V. parahaemolyticus*, and *V. vulnificus* could not be found, but resistances to beta-lactam antibiotics (ampicillin, cefazolin, oxacillin, and penicillin) and trimethoprim-sulfamethoxazole were detected. In addition, potentially human pathogenic *V. parahaemolyticus* and *V. vulnificus* could be identified according to their virulence-associated genes.

## Data Availability Statement

The original contributions presented in the study are included in the article/**Supplementary Material**. Further inquiries can be directed to the corresponding authors.

## Author Contributions

The research study was conceived by NB, SF, IH, and GR. Sample preparation and analysis were performed by SF, IH, JW, JS, and ES. The figures were provided by SF and IH. The manuscript was written by SF. All authors critically discussed the results, and revised and reviewed the manuscript.

## Funding

Support of this study was provided by the German Federal Environment Agency through Grant FKZ 3716 62 203 0.

## Conflict of Interest

The authors declare that the research was conducted in the absence of any commercial or financial relationships that could be construed as a potential conflict of interest.

## Publisher’s Note

All claims expressed in this article are solely those of the authors and do not necessarily represent those of their affiliated organizations, or those of the publisher, the editors and the reviewers. Any product that may be evaluated in this article, or claim that may be made by its manufacturer, is not guaranteed or endorsed by the publisher.

## References

[B1] Baker-Austin C. Oliver J. D. (2018). *Vibrio Vulnificus*: New Insights Into a Deadly Opportunistic Pathogen. Environ. Microbiol. 20 (2), 423–430. doi: 10.1111/1462-2920.13955 29027375

[B2] Baker-Austin C. Oliver J. D. Alam M. Ali A. Waldor M. K. Qadri F.. (2018). *Vibrio* Spp. Infections. Nat. Rev. Dis. Primers 4 (1), 8. doi: 10.1038/s41572-018-0005-8 30002421

[B3] Baker-Austin C. Stockley L. Rangdale R. Martinez-Urtaza J. (2010). Environmental Occurrence and Clinical Impact of *Vibrio Vulnificus* and *Vibrio Parahaemolyticus*: A European Perspective. Environ. Microbiol. Rep. 2 (1), 7–18. doi: 10.1111/j.1758-2229.2009.00096.x 23765993

[B4] Baker-Austin C. Trinanes J. Gonzalez-Escalona N. Martinez-Urtaza J. (2017). Non-Cholera Vibrios: The Microbial Barometer of Climate Change. Trends Microbiol. 25 (1), 76–84. doi: 10.1016/j.tim.2016.09.008 27843109

[B5] Baker-Austin C. Trinanes J. A. Salmenlinna S. Lofdahl M. Siitonen A. Taylor N. G. H.. (2016). Heat Wave-Associated Vibriosis, Sweden and Finlan. Emerg. Infect. Dis. 22 (7), 1216–1220. doi: 10.32032/eid2207.151996 27314874 PMC4918148

[B6] Baker-Austin C. Trinanes J. A. Taylor N. G. H. Hartnell R. Siitonen A. Martinez-Urtaza J. (2013). Emerging *Vibrio* Risk at High Latitudes in Response to Ocean Warming. Nat. Climate Change 3 (1), 73–77. doi: 10.1038/Nclimate1628

[B7] Bhat P. Bhaskar M. Sistla S. Kadhiravan T. (2019). Fatal Case of Necrotising Fasciitis Due to *Vibrio Vulnificus* in a Patient With Alcoholic Liver Disease and Diabetes Mellitus. BMJ Case Rep. 12 (1):e227851. doi: 10.1136/bcr-2018-227851 PMC6340558 30659010

[B8] Bier N. Bechlars S. Diescher S. Klein F. Hauk G. Duty O.. (2013). Genotypic Diversity and Virulence Characteristics of Clinical and Environmental *Vibrio Vulnificus* Isolates From the Baltic Sea Region. Appl. Environ. Microbiol. 79 (12), 3570–3581. doi: 10.1128/Aem.00477-13 23542621 PMC3675912

[B9] Bier N. Jackel C. Dieckmann R. Brennholt N. Boer S. I. Strauch E. (2015). Virulence Profiles of *Vibrio Vulnificus* in German Coastal Waters, a Comparison of North Sea and Baltic Sea Isolates. Int. J. Environ. Res. Public Health 12 (12), 15943–15959. doi: 10.3390/ijerph121215031 26694432 PMC4690967

[B10] Boer S. I. Heinemeyer E. A. Luden K. Erler R. Gerdts G. Janssen F.. (2013). Temporal and Spatial Distribution Patterns of Potentially Pathogenic Vibrio Spp. At Recreational Beaches of the German North Sea. Microb. Ecol. 65 (4), 1052–1067. doi: 10.1007/s00248-013-0221-4 23563708

[B11] Brehm T. T. Dupke S. Hauk G. Fickenscher H. Rohde H. Berneking L. (2021). Non-Cholera *Vibrio* Species - Currently Still Rare But Growing Danger of Infection in the North Sea and the Baltic Sea. Internist 62 (8), 876–886. doi: 10.1007/s00108-021-01086-x 34269833 PMC8283098

[B12] Bundesamt für Seeschifffahrt und Hydrographie, BSH (2020) Meerestemperaturen. Available at: https://www.bsh.de/DE/DATEN/Meerestemperaturen/Meeresoberflaechentemperaturen/meeresoberflaechentemperaturen_node.html (Accessed December 14, 2021).

[B13] Cantet F. Hervio-Heath D. Caro A. Le Mennec C. Monteil C. Quemere C.. (2013). Quantification of *Vibrio Parahaemolyticus, Vibrio Vulnificus* and *Vibrio Cholerae* in French Mediterranean Coastel Lagoons. Res. Microbiol. 164 (8), 867–874. doi: 10.1016/j.resmic.2013.06.005 23770313 PMC4073583

[B14] Ceccarelli D. Hasan N. A. Huq A. Colwell R. R. (2013). Distribution and Dynamics of Epidemic and Pandemic *Vibrio Parahaemolyticus* Virulence Factors. Front. Cell Infect. Microbiol. 3. doi: 10.3389/fcimb.2013.00097 PMC3858888 24377090

[B15] Centers for Disease Control and Prevention, CDC (2017) Vibrio Vulnificus Infections and Disasters. Available at: https://www.cdc.gov/disasters/vibriovulnificus.html (Accessed December 14, 2020).

[B16] Chowdhury G. Joshi S. Bhattacharya S. Sekar U. Birajdar B. Bhattacharyya A.. (2016). Extraintestinal Infections Caused by Non-Toxigenic *Vibrio Cholerae* non-O1/non-O139. Front. Microbiol. 7. doi: 10.3389/fmicb.2016.00144 PMC4749697 26904017

[B17] Deshayes S. Daurel C. Cattoir V. Parienti J. J. Quilici M. L. de la Blanchardiere A. (2015). Non-O1, non-O139 *Vibrio Cholerae* Bacteraemia: Case Report and Literature Review. Springerplus 4, 575. doi: 10.1186/s40064-015-1346-3 26543710 PMC4627963

[B18] European Centre for Disease Prevention and Control, ECDC (2020) Vibrio Risk Portal. E3 Geoportal. Available at: https://e3geoportal.ecdc.europa.eu/SitePages/Vibrio%20Map%20Viewer (Accessed December 14, 2021).

[B19] Froelich B. A. Ayrapetyan M. Fowler P. Oliver J. D. Noblea R. T. (2015). Development of a Matrix Tool for the Prediction of *Vibrio* Species in Oysters Harvested From North Carolina. Appl. Environ. Microbiol. 81 (3), 1111–1119. doi: 10.1128/Aem.03206-14 25452288 PMC4292490

[B20] Froelich B. Bowen J. Gonzalez R. Snedeker A. Noble R. (2013). Mechanistic and Statistical Models of Total *Vibrio* Abundance in the Neuse River Estuary. Water Res. 47 (15), 5783–5793. doi: 10.1016/j.watres.2013.06.050 23948561

[B21] Froelich B. A. Daines D. A. (2020). In Hot Water: Effects of Climate Change on *Vibrio-*Human Interactions. Environ. Microbiol. 22 (10), 4101–4111. doi: 10.1111/1462-2920.14967 32114705

[B22] Froelich B. Gonzalez R. Blackwood D. Lauer K. Noble R. (2019). Decadal Monitoring Reveals an Increase in *Vibrio* Spp. Concentrations in the Neuse River Estuary, North Carolina, USA. PLoS One 14 (4). doi: 10.1371/journal.pone.0215254 PMC6478372 31013284

[B23] Froelich B. Oliver J. D. (2013). The Interactions of *Vibrio Vulnificus* and the Oyster Crassostrea Virginica. Microb. Ecol. 65 (4), 807–816. doi: 10.1007/s00248-012-0162-3 23280497

[B24] Guillod C. Ghitti F. Mainetti C. (2019). *Vibrio Parahaemolyticus* Induced Cellulitis and Septic Shock After a Sea Beach Holiday in a Patient With Leg Ulcers. Case Rep. Dermatol. 11 (1), 94–100. doi: 10.1159/000499478 31123452 PMC6514507

[B25] Han F. Ge B. (2010). Multiplex PCR Assays for Simultaneous Detection and Characterization of *Vibrio Vulnificus* Strains. Lett. Appl. Microbiol. 51 (2), 234–240. doi: 10.1111/j.1472-765X.2010.02887.x 20586937

[B26] Hendren N. Sukumar S. Glazer C. S. (2017). *Vibrio Vulnificus* Septic Shock Due to a Contaminated Tattoo. BMJ Case Rep., 2017 1–2. doi: 10.1136/bcr-2017-220199 PMC5614220 28551603

[B27] Huehn S. Eichhorn C. Urmersbach S. Breidenbach J. Bechlars S. Bier N.. (2014). Pathogenic *Vibrios* in Environmental, Seafood and Clinical Sources in Germany. Int. J. Med. Microbiol. 304 (7), 843–850. doi: 10.1016/j.ijmm.2014.07.010 25129553

[B28] Huq A. Sack R. B. Nizam A. Longini I. M. Nair G. B. Ali A.. (2005). Critical Factors Influencing the Occurrence of *Vibrio Cholerae* in the Environment of Bangladesh. Appl. Environ. Microbiol. 71 (8), 4645–4654. doi: 10.1128/Aem.71.8.4645-4654.2005 16085859 PMC1183289

[B29] Intergovernmental Panel on Climate Change, IPCC (2019) Impacts of 1.5°C of Global Warming on Natural and Human Systems. Available at: https://www.ipcc.ch/site/assets/uploads/sites/2/2019/06/SR15_Chapter3_Low_Res.pdf (Accessed December 14, 2021).

[B30] Jones M. K. Oliver J. D. (2009). *Vibrio Vulnificus*: Disease and Pathogenesis. Infect. Immun. 77 (5), 1723–1733. doi: 10.1128/IAI.01046-08 19255188 PMC2681776

[B31] Kaneko T. Colwell R. R. (1973). Ecology of *Vibrio Parahaemolyticus* in Chesapeake Bay. J. Bacteriol. 113 (1), 24–32. doi: 10.1128/jb.113.1.24-32.1973 4567138 PMC251597

[B32] Kishishita M. Matsuoka N. Kumagai K. Yamasaki S. Takeda Y. Nishibuchi M. (1992). Sequence Variation in the Thermostable Direct Hemolysin-Related Hemolysin (Trh) Gene of *Vibrio Parahaemolyticus* . Appl. Environ. Microbiol. 58 (8), 2449–2457. doi: 10.1128/Aem.58.8.2449-2457.1992 1514791 PMC195802

[B33] Lee C. Y. Cheng M. F. Yu M. S. Pan M. J. (2002). Purification and Characterization of a Putative Virulence Factor, Serine Protease, From *Vibrio Parahaemolyticus* . FEMS Microbiol. Lett. 209 (1), 31–37. doi: 10.1111/j.1574-6968.2002.tb11105.x 12007650

[B34] Leng F. Lin S. Wu W. Zhang J. Song J. Zhong M. (2019). Epidemiology, Pathogenetic Mechanism, Clinical Characteristics, and Treatment of *Vibrio Vulnificus* Infection: A Case Report and Literature Review. Eur. J. Clin. Microbiol. Infect. Dis. 38 (11), 1999–2004. doi: 10.1007/s10096-019-03629-5 31325061

[B35] Makino K. Oshima K. Kurokawa K. Yokoyama K. Uda T. Tagomori K.. (2003). Genome Sequence of *Vibrio Parahaemolyticus*: A Pathogenic Mechanism Distinct From That of *V Cholerae* . Lancet 361 (9359), 743–749. doi: 10.1016/S0140-6736(03)12659-1 12620739

[B36] Mantri C. K. Mohapatra S. S. Ramamurthy T. Ghosh R. Colwell R. R. Singh D. V. (2006). Septaplex PCR Assay for Rapid Identification of *Vibrio Cholerae* Including Detection of Virulence and Int SXT Genes. FEMS Microbiol. Lett. 265 (2), 208–214. doi: 10.1111/j.1574-6968.2006.00491.x 17081197

[B37] Martinez-Urtaza J. Bowers J. C. Trinanes J. DePaola A. (2010). Climate Anomalies and the Increasing Risk of *Vibrio Parahaemolyticus* and *Vibrio Vulnificus* Illnesses. Food Res. Int. 43 (7), 1780–1790. doi: 10.1016/j.foodres.2010.04.001

[B38] Metelmann C. Metelmann B. Grundling M. Hahnenkamp K. Hauk G. Scheer C. (2020). *Vibrio Vulnificus*, an Increasing Threat of Sepsis in Germany? Anaesthesist 69 (9), 672–678. doi: 10.1007/s00101-020-00811-9 32620988

[B39] National Centers for Environmental Information, NOAA (2010-2020) Climate at a Glance: National Time Series. Available at: https://www.ncdc.noaa.gov/cag/ (Accessed March 03, 2020).

[B40] Nigro O. D. Hou A. X. Vithanage G. Fujioka R. S. Steward G. F. (2011). Temporal and Spatial Variability in Culturable Pathogenic *Vibrio* Spp. in Lake Pontchartrain, Louisiana, Following Hurricanes Katrina and Rita. Appl. Environ. Microbiol. 77 (15), 5384–5393. doi: 10.1128/Aem.02509-10 21642406 PMC3147459

[B41] Nishibuchi M. Fasano A. Russell R. G. Kaper J. B. (1992). Enterotoxigenicity of *Vibrio Parahaemolyticus* With and Without Genes Encoding Thermostable Direct Hemolysin. Infect. Immun. 60 (9), 3539–3545. doi: 10.1128/Iai.60.9.3539-3545.1992 1500161 PMC257358

[B42] Oliver J. D. Pruzzo C. Vezzulli L. Kaper J. B. (2013). “Vibrio Species,” in Food Microbiology: Fundamentals and Frontiers, 4th edn. Eds. Doyle M. P. Buchanan R. L. (Washington: ASM), pp 401–pp 440. doi: 10.1128/9781555818463.ch16

[B43] Park K. S. Ono T. Rokuda M. Jang M. H. Okada K. Idia T.. (2004). Functional Characterization of Two Type III Secretion Systems of *Vibrio Parahaemolyticus* . Infect. Immun. 72 (11), 6659–6665. doi: 10.1128/Iai.72.11.6659-6665.2004 15501799 PMC523034

[B44] Pruzzo C. Huq A. Colwell R. R. Donelli G. (2005). “Pathogenic Vibrio Species in the Marine and Estuarine Environment,” in Oceans and Health: Pathogens in the Marine Environment (Heidelberg: Springer), 217–252. doi: 10.1007/0-387-23709-7_9

[B45] Randa M. A. Polz M. F. Lim E. (2004). Effects of Temperature and Salinity on *Vibrio Vulnificus* Population Dynamics as Assessed by Quantitative PCR. Appl. Environ. Microbiol. 70 (9), 5469–5476. doi: 10.1128/Aem.70.9.5469-5476.2004 15345434 PMC520858

[B46] Restrepo D. Huprikar S. S. VanHorn K. Bottone E. J. (2006). O1 and non-O1 *Vibrio Cholerae* Bacteremia Produced by Hemolytic Strains. Diagn. Microbiol. Infect. Dis. 54 (2), 145–148. doi: 10.1016/j.diagmicrobio.2005.08.008 16426794

[B47] Schirmeister F. Dieckmann R. Bechlars S. Bier N. Faruque S. M. Strauch E. (2014). Genetic and Phenotypic Analysis of *Vibrio Cholerae* non-O1, non-O139 Isolated From German and Austrian Patients. Eur. J. Clin. Microbiol. Infect. Dis. 33 (5), 767–778. doi: 10.1007/s10096-013-2011-9 24213848 PMC3996285

[B48] Shin O. S. Tam V. C. Suzuki M. Ritchie J. M. Bronson R. T. Waldor M. K.. (2011). Type III Secretion is Essential for the Rapidly Fatal Diarrheal Disease Caused by non-O1, non-O139 *Vibrio Cholerae* . mBio 2 (3), e00106–e00111. doi: 10.1128/mBio.00106-11 21673189 PMC3111608

[B49] State Agency for Health and Social Affairs Mecklenburg-Western Pomerania, LAGuS (2014-2020) Pressemitteilung. Available at: https://www.lagus.mv-regierung.de/Services/Aktuelles/?id=140795&processor=processor.sa.pressemittelung (Accessed December 14, 2021).

[B50] Vezzulli L. Baker-Austin C. Kirschner A. Pruzzo C. Martinez-Urtaza J. (2020). Global Emergence of Environmental non-O1/O139 *Vibrio Cholerae* Infections Linked With Climate Change: A Neglected Research Field? Environ. Microbiol. 22 (10), 4342–4355. doi: 10.1111/1462-2920.15040 32337781

[B51] Vezzulli L. Colwell R. R. Pruzzo C. (2013). Ocean Warming and Spread of Pathogenic *Vibrios* in the Aquatic Environment. Microb. Ecol. 65 (4), 817–825. doi: 10.1007/s00248-012-0163-2 23280498

[B52] Vezzulli L. Grande C. Reid P. C. Helaouet P. Edwards M. Hofle M. G.. (2016). Climate Influence on *Vibrio* and Associated Human Diseases During the Past Half-Century in the Coastal North Atlantic. Proc. Natl. Acad. Sci. U.S.A. 113 (34), E5062–E5071. doi: 10.1073/pnas.1609157113 27503882 PMC5003230

[B53] Wetz J. J. Blackwood A. D. Fries J. S. Williams Z. F. Noble R. T. (2008). Trends in Total *Vibrio* Spp. And *Vibrio Vulnificus* Concentrations in the Eutrophic Neuse River Estuary, North Carolina, During Storm Events. Aquat. Microb. Ecol. 53 (1), 141–149. doi: 10.3354/ame01223

[B54] Yang C. Zhang X. Fan H. Li Y. Hu Q. Yang R.. (2019). Genetic Diversity, Virulence Factors and Farm-to-Table Spread Pattern of *Vibrio Parahaemolyticus* Food-Associated Isolates. Food Microbiol. 84, 103270. doi: 10.1016/j.fm.2019.103270 31421783

