# Supplementary figures and images for: Prevalence and Distribution of Potentially Human Pathogenic Vibrio spp. on German North and Baltic Sea Coasts
Source: Front Cell Infect Microbiol. 2022 Jul 22;12:846819. doi: 10.3389/fcimb.2022.846819 (PMC9355094; doi:10.3389/fcimb.2022.846819)

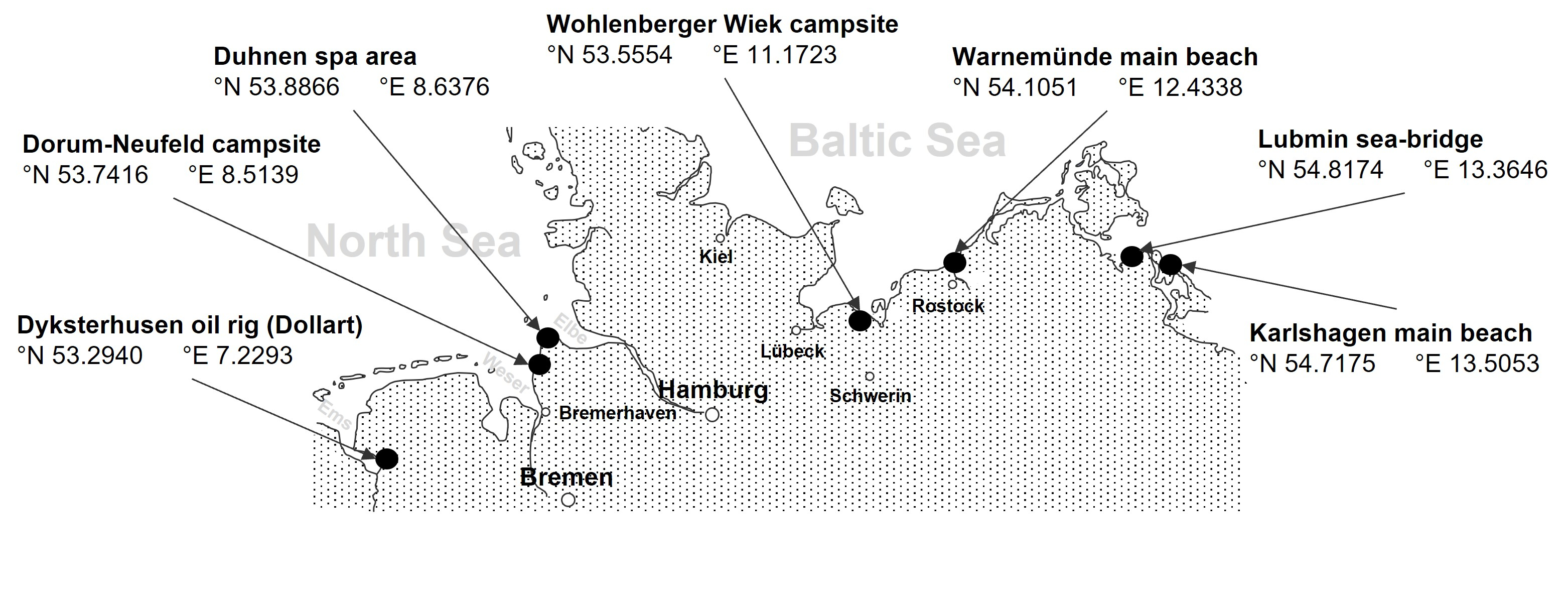

Supplement: Supplementary file 1 [file Image_1.jpeg]
